# Pyrokinin β-Neuropeptide Affects Necrophoretic Behavior in Fire Ants (*S. invicta*), and Expression of β-NP in a Mycoinsecticide Increases Its Virulence

**DOI:** 10.1371/journal.pone.0026924

**Published:** 2012-01-04

**Authors:** Yanhua Fan, Roberto M. Pereira, Engin Kilic, George Casella, Nemat O. Keyhani

**Affiliations:** 1 Biotechnology Research Center, Southwest University, Beibei, Chongqing, People's Republic of China; 2 Department of Entomology and Nematology, University of Florida, Gainesville, Florida, United States of America; 3 Department of Microbiology and Cell Science, University of Florida, Gainesville, Florida, United States of America; University of Wisconsin – Madison, United States of America

## Abstract

Fire ants are one of the world's most damaging invasive pests, with few means for their effective control. Although ecologically friendly alternatives to chemical pesticides such as the insecticidal fungus *Beauveria bassiana* have been suggested for the control of fire ant populations, their use has been limited due to the low virulence of the fungus and the length of time it takes to kill its target. We present a means of increasing the virulence of the fungal agent by expressing a fire ant neuropeptide. Expression of the fire ant (*Solenopsis invicta*) pyrokinin β -neuropeptide (β-NP) by *B. bassiana* increased fungal virulence six-fold towards fire ants, decreased the LT_50_, but did not affect virulence towards the lepidopteran, *Galleria mellonella*. Intriguingly, ants killed by the β-NP expressing fungus were disrupted in the removal of dead colony members, i.e. necrophoretic behavior. Furthermore, synthetic C-terminal amidated β-NP but not the non-amidated peptide had a dramatic effect on necrophoretic behavior. These data link chemical sensing of a specific peptide to a complex social behavior. Our results also confirm a new approach to insect control in which expression of host molecules in an insect pathogen can by exploited for target specific augmentation of virulence. The minimization of the development of potential insect resistance by our approach is discussed.

## Introduction

The spread of fire ants is considered a classic example of world-wide biological invasions of a species into previously unoccupied habitats with the potential to result in significant ecosystem alterations. The red imported fire ant (*Solenopsis invicta*), native to South America, is considered by the World Conservation Unit as one of the top 100 worst invasive alien species, and its detrimental impact on humans, domestic and wild animals, agriculture, and ecosystems is well-documented [1], [2], [3]. It is a major invasive pest insect to almost the entire Southeastern United States and continues to expand it range north and westwards causing agricultural and ecosystem disruptions that extend from crop losses to declines of native species [4]. Fire ant have continued to spread despite the treatment of over 56 million hectares with Mirex bait alone and tons of other chemical insecticides [5], which themselves have significant damaging environmental consequences. Biological control of fire ants using entomopathogenic fungi, such as *Beauveria bassiana*, offers a more environmentally friendly alternative to chemical pesticides [6], [7], [8], [9]. The use of entomopathogenic fungi, however, has met with limited success partially due to the relatively long time (3–10 days) it can take for the fungus to kill target insects. Ants have posed a particular challenge due to communal behaviors such as grooming and nest cleaning which can decrease the efficacy of microbial agents [10]. Previous work has shown that the potency of fungal insecticides can be improved [11]. Expression of a 70 amino acid scorpion (*Androctonus australis*)-derived neurotoxin in the fungal insect pathogen, *Metarhizium anisopliae*, increased its toxicity 9-fold against *Aedes aegyptii* as compared to its wild-type parent [12]. Here, we sought to use a different approach, namely to express host molecules, e.g. hormones or neuropeptides, in the fungal pathogen. As the fungus targets the insect, it will produce the host molecule, disrupting the normal endocrine or neurological balance of the host. The desired outcome is to make the target (fire ant) more susceptible to the invading fungus, thus increasing the potency of the fungal agent. As candidates for expression in the fungus we sought to use a recently described strategy whereby peptides that participate in a critical host physiological process are used [13]. Depending upon the molecule (peptide) chosen, in theory, the increased virulence can, to a particular degree, be host specific, thus minimizing non-target effects.

The pyrokinin/pheromone biosynthesis activating neuropeptide (PBAN) family consists of insect neurohormones characterized by the presence of a C-terminal FXPRL amine sequence [14], [15]. First isolated from the cockroach, *Leucophaea maderae*, as a myotropic (visceral muscle contraction stimulatory) peptide, members of this peptide family are widely distributed within the Insecta, where depending upon the species, they function in a diverse range of physiological processes that includes stimulation of pheromone biosynthesis, melanization, acceleration of pupariation, and induction and/or termination of diapause [16], [17], [18]. In the natural insect host, these peptides are C-terminal amidated, a modification often required for their activity. In Lepidoptera, the PBAN peptide is encoded on a translated ORF that is subsequently processed (cleaved) to yield diapause hormone (DH), and the α-, β-, and γ-neuropeptides, along with the PBAN peptide itself (which is found between the β- and γ-neuropeptides). More recently, isolation of a cDNA sequence for the fire ant, *S. invicta*, led to the identification of PBAN and related peptide homologs [19]. Analysis of the ORF revealed the presence of DH, as well β- and γ-neuropeptide homologs, but no α-neuropeptide.

Here, we assessed the impact of expressing the β-NP peptide in the fungal insect pathogen *B. bassiana*. Our data show a decrease in both the lethal dose (LD_50_) and lethal time (LT_50_) it takes to kill target fire ants in the β-NP expressing strain as compared to its wild-type parent. The effect was host specific, and no increase in virulence was noted when the strain was tested against the greater wax moth, *Galleria mellonella*. By using a host molecule the chances of resistance are minimized due to the simple fact that the fungal-expressed peptide represents a host molecule that is regulated in both tissue specific and developmental patterns. Any mutations that could compensate for the increased dose given by the fungus during infection would be significantly compromised, indeed, potentially dependent upon the fungus for proper development. Unexpectedly, we observed that the cadavers of ants killed by the β-NP expressing *B. bassiana* strain were treated differently, i.e. removed slower, than controls or those killed by the WT fungus. Experiments testing the effects of synthetic peptides on cadaver removal or necrophoretic behavior resulted in another serendipitous result, namely that ant cadavers treated with the β-NP-NH_2_ peptide were removed much more rapidly than β-NP or control treated cadavers. The implications of these results in terms of biological control of ants and chemical sensing are discussed.

## Materials and Methods

### Construction of expression vector and fungal transformation

The *S. invicta* pyrokinin β-neuropeptide (β-NP, QPQFTPRL) was fused to a 28-amino acid signal peptide derived from the *B. bassiana* chitinases-1 (*chit1*) gene [20] and cloned under control of the *B. bassiana* glyceraldehyde phosphate dehydrogenase promoter (P_gpd-Bb_). Primer pairs P1/P2 (5′-GTTGGGTATGCTCCGGCGCG, & 5′-GGTTGTTATTGATTAAAAGG) were used to amplify P_gpdA-Bb_ using *B. bassiana* genomic DNA as templates. The *B. bassiana* chitinase (*Bbchit1*) derived signal peptide (SP) was obtained with the primer pair P3/P4; (P3, 5′-CCTTTTAATCAATAACAACCATGGCTCCTTTTCTTCAAAC & P4, 5′- TTAGAGGCGGGGGGTAAACTGGGGCTGTCGCGGCGCCAAGGGCGAGG) using *B. bassiana* genomic DNA as the template and with the β-NP coding sequence incorporated into primer P4. These primers were designed containing a 20 bp overlap sequences between P_gpdA-Bb_ and SP: β-NP. The desired construct (P_gpdA-Bb_:β-NP) was produced via primer-less assembly in a reaction mixture containing: 5 µl 5×Phusion Taq polymerase buffer, 2 µl 2.5 mM dNTP, 30 ng P_gpdA-Bb_, 30 ng SP: β-NP, 0.4 U Phusion Taq DNA polymerase, total volume 25 µl. PCR reaction cycling conditions: 98°C (2 min); followed by 25 cycles of: 98°C (20 s), 56°C (30 sec), 72 (1 min); and 72°C (5 min). Primer pair P1 & P4 were used to obtain the P_gpdA-Bb_:*SP-* β-NP fragment using the assembled product as template. The obtained fragments were cloned into pDrive vector (Qiagen) and verified by sequencing. P_gpdA-Bb_:SP-β-NP was subcloned from pDrive vector via *EcoR*I restriction sites into pUC-Bar, yielding pUC-Bar-P_gpdA-Bb_:SP-β-NP. This plasmid was linearized with *Xba*I and transformed into *B. bassiana* competent cells as described [21]. The resultant strain was labeled Bb::spβ-NP_gpd_.

### Purification and identification of β-NP from fungi cultures using HPLC and MS/MS

In order to verify (extracellular) β-NP production in the recombinant *B. bassiana* strain, fungal cultures (Bb::spβ-NP_gpd_ and the WT parent) were grown first grown in SDBY (Sabouraud dextrose broth with 0.5% yeast extract) for 2 d, after which 1.5 g of washes cells were transferred to Czapek-dox broth (50–100 ml) for 3 days. Fungal cells were removed by centrifugation, the resultant supernatant filtered through a 0.22 µm filter, and the supernatant samples subsequently lyophilized and stored at −20°C until used. Lyophilized samples were rehydrated in 3.0 ml of water containing 0.1% TFA, and applied onto a C_18_ reverse phase SepPak column. The column was washed with 0.1% TFA and peptides were eluted with 80% acetonitrile-0.1% TFA. The eluted fraction (in acetonitrile) was dried in a SpeedVac, resuspended in water-0.1% TFA (0.5 ml) and chromatographed on a C_18_ reversed phase HPLC column with eluting factions monitored via absorbance at 214 nm. Fractions eluting at the same retention time as an initial run using synthetic β-NP used as a standard, were collected, dried with a fine stream of N_2_, rehydrated to 0.2 ml with water-0.1% TFA, and rechromatographed as above. Fractions were collected as above, dried under N_2_ and analyzed by LC-MS/MS (University of Florida, Dept. of Chemistry, analytical Services). A standard curve using synthetic β-NP was made in order to quantify the amount of peptide in the sample.

### Insect Bioassays

*S. invicta* colonies were collected from the field, separated from the soil by drip flotation and maintained in Fluon-coated trays with a diet consistaing of 10% sucrose solution, a variety of freeze-killed insects, fruits and vegetables, and chicken eggs. Fungal cultures were grown on potato dextrose agar (PDA). Plates were incubated at 26°C for 14–21 d, and aerial conidia (spores) were harvested by flooding or scraping the plates with sterile distilled H_2_O containing 0.05% Tween 80. Spores concentrations were determined by direct count using a hemocytometer and adjusted to the desired concentration for use (typically between 10^6^–10^8^ conidia/ml). Two types of bioassays were used to assess the virulence of the fungal strains: (1) “classical bioassay” using *S. invicta* workers. Test groups of ants (25/chamber) were inoculated with fungal suspensions (concentrations ranging from 10^6^–10^8^ conidia/ml) using a spray tower as described [22]. The ants were housed in plastic cups (ø = 6 cm) whose sides had been coated with Fluon and topped with a perforated lid. Ants were given 10% sucrose solutions in 1.5 ml Eppendorf tubes with a cotton plug. Experiments were performed at 26°C and mortality was recorded daily. Controls were treated with Tween-80 and the mortality assays were repeated at least three times. (2) “Mock mini-mound” assays. Larger scale bioassays were performed using larger test chambers (ø = 19 cm). Test chambers contained a small Petri dish (ø = 3 cm) containing moist dental plaster, that served as the nest for the mini-mound. Ants (0.5 gm, ∼2,000 individuals) including 3–4 dealate reproductive females were placed in the test chamber that included a 10% sucrose solution in an Eppendorf tube. Treatments and assay conditions were identical to the classical bioassay. Duplicate samples were performed for each experiment and the entire assay repeated three times with independent batches of fungal spores. For all experiments, a *χ^2^*-test was first used to determine homogeneity among variance of the repeats (*p*<0.05). Further statistical analysis of the mortality was performed using SPSS which was used to estimate the median lethal time (LT_50_), the median lethal concentration (LC_50_), fiducial limits and other regression parameters.

### Necrophoretic behavior assays

Assay chambers and methods were based upon a previously described protocol [23]. Briefly, the conical end of a 15 ml polypropylene tube (nest) was cut off and connected via a short tubing (ø = 8 mm, 10 cm long) to a round plastic container (ø = 19 cm, foraging arena) into which a hole had been punched out in the bottom at the middle of the container. Test ants (0.1 gm, ∼400 ants with at least one dealate) were placed in the assay chamber and allowed to equilibrate for 1–2 hr before the experiment was initiated. Three separate experimental protocols were employed. (1) Freeze killed; ants killed by the WT *B. bassiana* strain, and ants killed by the Bb::spβ-NP_gpd_ strain were presented to untreated ants. For the freeze-killed ants, ants were placed at −80°C for 15 min, and then placed at R.T. for 24 hr before use. For fungal-killed ants, infections were performed as described above and the dead ants removed daily. Test ants were derived from those that died on day 4 post-infection. To measure necrophoretic behavior, the test items (5–10 dead ants) were placed in a ring around (1 cm from) the nest entrance. The time interval between introduction and removal of each item was recorded up to a time limit of 600–800 minutes. The number of test objects that were not moved within this interval was also recorded. (2) The effect of infection on necrophoretic behavior was probed by presenting WT- or Bb::spβ-NP_gpd_-killed ants to (a) uninfected ants, (b) WT-infected ants, or (c) Bb::spβ-NP_gpd_-infected ants. Ants were infected (5×10^7^ conidia/ml) with the fungal strains 2 d prior to testing. Test objects (dead ants) were prepared and tested as described above. (3) The effect of synthetic peptides on ant cadaver removal was evaluated by having three peptides; (a) β-NP (QPQFTPRL, no C-terminal amidation), (b) β-NP-NH_2_ (QPQFTPRL-NH_2_), and (c) QAGVTGHA-NH_2_ (control 8 amino acid amidated peptide) synthesized (GenScript, Piscataway, NJ). Freeze-killed ants (15 min at −80°C, allowed to thaw for 15 min R.T.) were immersed in 100 nM solutions (resuspended in sterile distilled H_2_O) of the test peptide or H_2_O alone for 30 s, and then allowed to air-dry for 30 min on a Kim-wipe towel. Necrophoretic behavior to the ants was measured as described above. P-values were obtained from an analysis of variance (1 or 2 way-ANOVA) for each data set, using a permutation test to guard against possible non-normality. 10,000 permutations were used for each test statistic. The unknown (i.e. never moved test objects) data had no effect on the analysis.

## Results

### Construction and bioassay of β-NP expressing *B. bassiana*

The fire ant β-NP, comprised of the eight-amino acid sequence, QPQFTPRL, was expressed in *B. bassiana* via transformation of an expression vector containing a constitutive *B. bassiana*-derived gpd-promoter, and the nucleotide sequence corresponding to the β-NP peptide fused to a 28-amino acid signal sequence derived from the *B. bassiana* chitinase (*chit1*) gene to produce strain Bb::spβ-NP_gpd_. Heterologous expression of the peptide was confirmed by partial purification and mass spectrometry analysis of culture supernatants. These data indicated the production of a non-amidated β-NP peptide by the fungus at a concentration of ∼0.2–0.4 µM.

**Figure 1:**
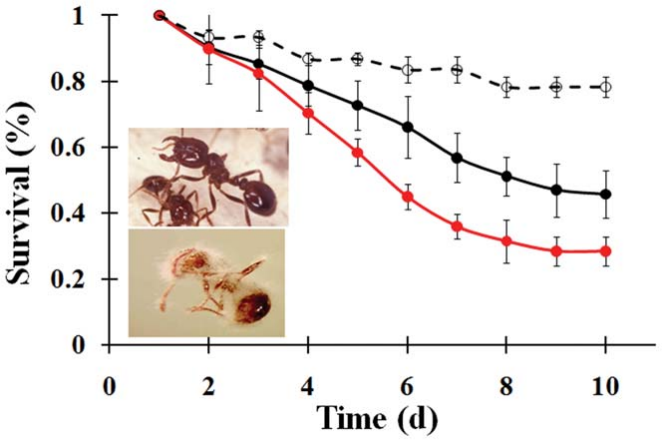
Fire ant bioassays infected with either the WT (•, black line) or Bb::spβ-NP_gpd_ (•, red line) *B. bassiana* strains and buffer treated controls (○, dashed line). The percent survival of *S. invicta* treated with 4×10^6^ conidia/ml of each strain over the indicated time course is presented. These data were used to calculate the LT_50_ values, and a concentration curve was used to determine the LD_50_ values. Inset, top panel, normal uninfected ant; bottom panel, *B. bassiana* infected ant (14 d).

Both classical worker group and mock mound assays were used to assess the virulence of WT and β-NP expressing *B. bassiana* strains. Bb::spβ-NP_gpd_ was much more potent (P<0.001) than WT, causing 50% mortality against fire ants after 5 days post-infection with an LD_50_ of 1.5±0.9×10^7^ conidia/ml compared to an LD_50_ of 1.0±0.7×10^8^ conidia/ml for the WT parent. Thus, it takes 6–7-fold fewer conidia to provide the same level of mortality. Expressing β-NP also significantly reduced survival times (Fig. 1). At a concentration of 2×10^7^ conidia/ml, the mean lethal time to achieve 50% mortality (LT_50_) was reduced from 177±11 hr for the WT to 122±5 hr for the β-NP expressing strain; representing ∼30% reduction in the mean survival time (P<0.01). At lower spore concentrations (4×10^6^ conidia/ml) the effect was even more dramatic, with the WT LT_50_ reaching 211±23 hr and the β-NP expressing strain 135±7 hr (P<0.001). In order to determine whether expression of the fire ant β-NP would affect virulence towards other insect, bioassays were performed with several other insect species. No significant difference was noted between the virulence of the WT and Bb::spβ-NP_gpd_ strains towards the lepidopteran host, *Galleria mellonella*, in which the LT_50_ values were 158±5 hr and 166±8 hr, for the WT and β-NP expressing strains, respectively (P>0.05). Similarly, no difference was noted between the WT and β-NP expressing strains when tested against the tobacco hornworm, *Manduca sexta*.

### Alternations in ant social behavior mediated by β-NP

**Figure 2:**
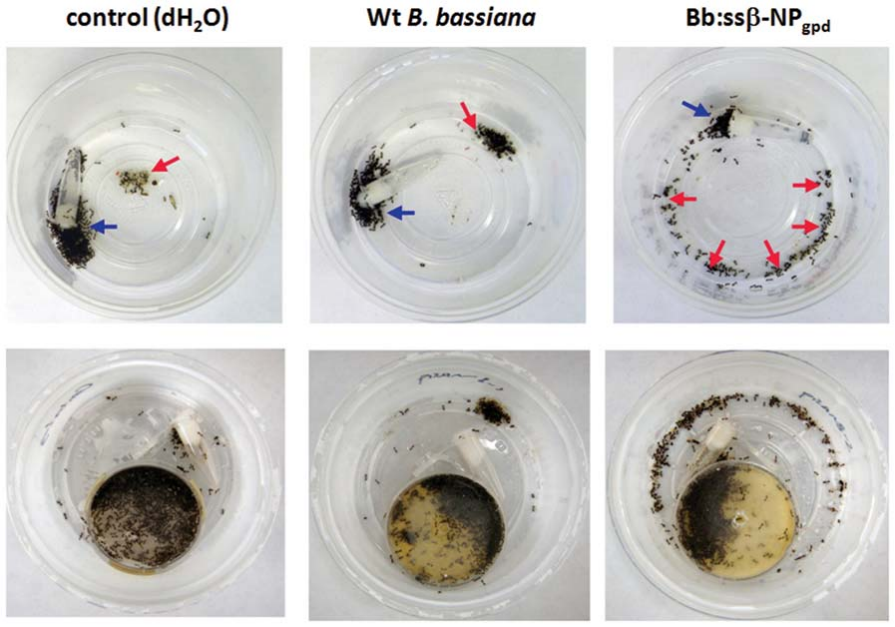
Distribution of dead ants after infection with WT and Bb::spβ-NP_gpd_ *B. bassiana* strains in mock mound assays. Top panels, ants in arenas with 10% sucrose, but no nest area, bottom panels, test arenas containing 10% sucrose and nest area. Blue arrows in top panels indicate the location of the majority of live ants, in the bottom panels, the most of the live ants are in the nest area (small petri dish in test arena). Red arrows indicate the presence of well defined “bone piles” in the control and WT *B. bassiana* infected ants, and a more random distribution of dead ants in the β-NP expressing *B. bassiana* infected ants.

**Figure 3:**
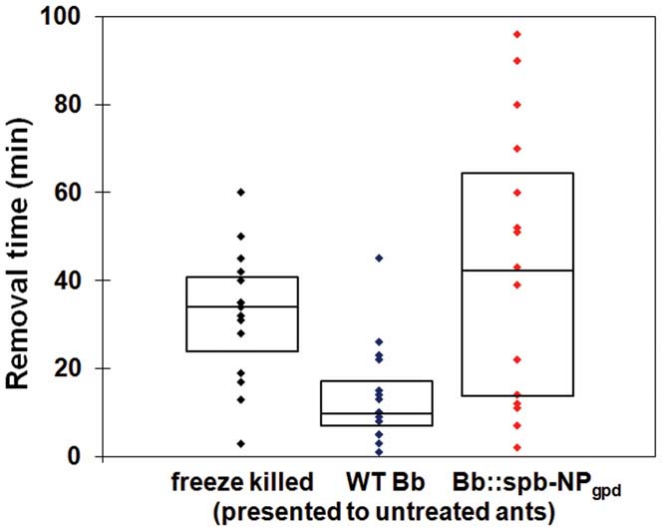
Responses of *S. invicta* to dead ants. Plots of the times between introduction and removal of items in the test arena. Boxes are bounded by the first quartile, median, and third quartile. Movement times of freeze-killed and fungal-killed, WT and Bb::spβ-NP_gpd_, ants presented to untreated ants. WT-killed ants were moved significantly more quickly than either freeze-killed or Bb::spβ-NP_gpd_-killed ants (P = 0.0014).

**Figure 4:**
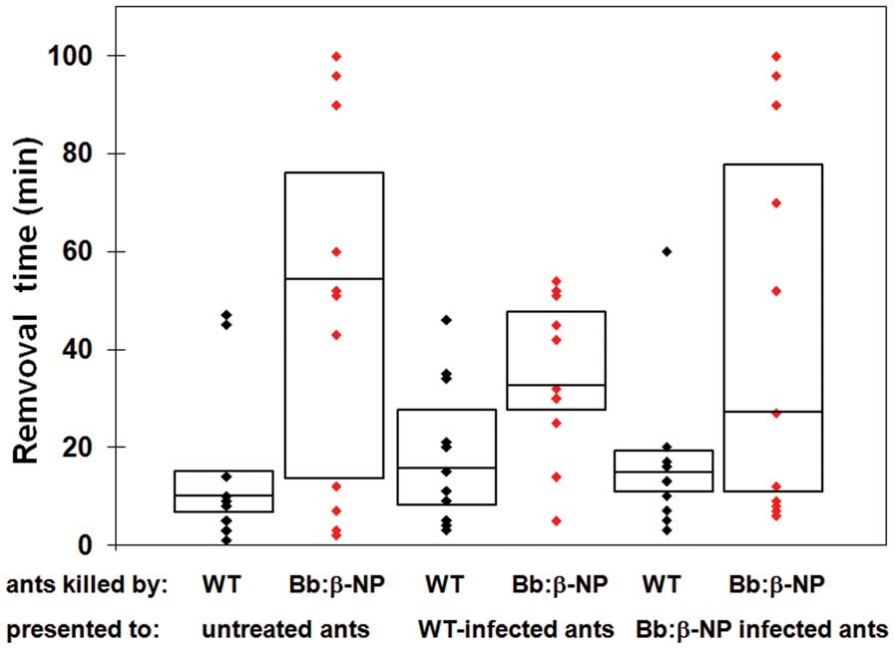
Movement times of WT- or Bb::spβ-NP_gpd_-killed ants by untreated ants, WT-infected (2 d prior to assay) ants, or Bb::spβ-NP_gpd_-infected ants. No significant differences were noted between live ants treatments (P = 0.86), with WT-killed ants moved significantly faster than Bb::spβ-NP_gpd_-killed ants regardless of the tested infection state of the ants doing the moving (P<0.001). This difference was consistent over the live ant treatments (interaction P-value = 0.53).

In the course of performing mock fire ant mound experiments we noted that ants infected with the Bb::spβ-NP_gpd_ strain appeared altered in their necrophoretic, or disposal of the dead, behavior (Fig. 2). Whereas mock-treated and WT *B. bassiana*-infected ants disposed of their dead in well defined “bone piles”, Bb::spβ-NP_gpd_-infected ants appeared to have randomly scattered piles of dead throughout the assay chamber, although typically at the periphery. In order to further probe this observation, we examined the responses of workers to nestmate corpses by placing corpses near the nest entrance in an experimental arena, and monitoring the time taken to remove the corpses. Workers moved ants killed by the WT *B. bassiana* strain faster than freeze-killed ants (∼24 hr old, P<0.01), but not those killed by the Bb::spβ-NP_gpd_ strain, which showed a wider variation, but was not significantly different from the response to the freeze-killed ants (Fig. 3). Thus, expression of the β-NP peptide appeared to delay removal of corpses. The large variation in removal time observed with Bb::spβ-NP_gpd_-infected ants may be due to differences in levels of β-NP expression in infected ants resulting from differential fungal growth within individual ant hosts. The infection state of the ants themselves did not appear to make a significant difference (P = 0.86). When WT or Bb::spβ-NP_gpd_-infected ants were presented with either WT- or Bb::spβ-NP_gpd_-killed ants, they moved the Bb::spβ-NP_gpd_-killed ants more slowly than WT-killed ones (P<0.001, Fig. 4). These experiments confirmed that ants killed by Bb::spβ-NP_gpd_ were treated differently than WT-killed ants, which were more rapidly removed regardless of the infection state of the ants themselves. This finding has potentially important application consequences since it may increase the lethality of the fungus in field applications due to reduced removal of cadavers which would increase the contact time and possible dispersal of the fungal agent within mounds.

**Figure 5:**
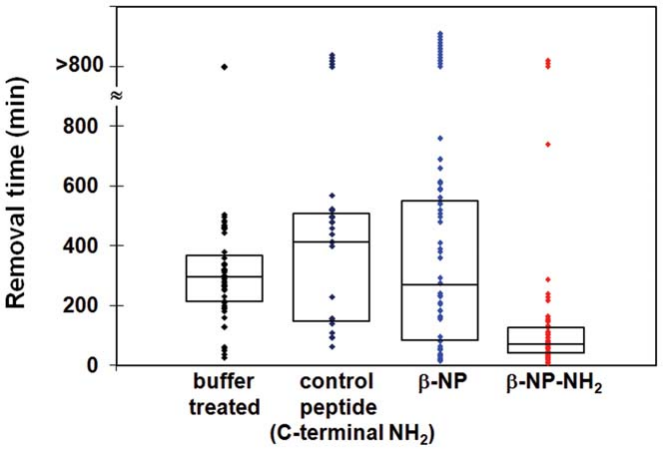
Movement times of dead ants treated with synthetic peptides and presented to untreated ants. Freeze-killed ants were immersed in 100 nM solution of either a control amidated peptide (QAGVTGHA-NH_2_), β-NP (QPQFTPRL), or β-NP-NH_2_ and presented to untreated ants. β-NP-NH_2_-treated ants were moved significantly faster than the control and other peptide treatments (P<0.001).

In order to further probe the effects of β-NP, a series of synthetic peptides were examined. Since both pheromonotropic and myotropic activity of pyrokinin/PBAN peptides have been demonstrated via topical application of the peptides onto insects [24], we sought to determine the effects of β-NP-NH_2_ (C-terminal amidated), β-NP (non-amidated peptide), and a control amidated peptide (QAGVTGHA-NH_2_) on the necrophoretic behavior of the fire ants. Freeze-killed ants were immersed in a 100 nM solution of the tested synthetic peptides and presented to untreated ants. Surprisingly, we found that ant corpses treated with the β-NP-NH_2_ peptide were moved significantly faster than buffer treated, β-NP-treated ants, or ants treated with a control eight-amino acid amidated peptide (P<0.001, Fig. 5). β-NP-treated ants were not moved any slower than control or buffer treated ants, although their distribution and the number of ants that were never removed within our assay conditions was larger for the β-NP treatment than for any other treatment examined.

## Discussion

The reconstruction of the global invasion history of fire ants, from introduction into the United States from their native South American range to their subsequent spread to newly colonized habitats worldwide, has highlighted the unintended perils and risks associated with the interconnected nature of global trade and travel [25]. The destructive nature of fire ant establishment and spread into new ecosystems has led to intense efforts at their control or eradication, in which, even fire ant detecting dogs have been employed [26]. The use of chemical pesticides has failed to stem the spread of fire ants, resulted in the emergence of pesticide resistance, and has not been without controversy [5], [27]. Thus, there has been much interest in the use of biological control strategies for fire ant control ranging from release of various parasites including mites and phorid flies, to the use of viruses, microsporidia, nematodes, and fungi [6]. The use entomopathogenic fungi, such as *B. bassiana*, although promising in several studies, has thus far met with limited success [6], [28], [29]. Although newer formulation technologies have increased their field efficacy, the relatively slow kill rate of these fungi coupled to ant behavioral responses such as grooming and corpse removal continue to pose significant obstacles to the use of entomopathogenic fungi [10], [30].

Recent efforts have demonstrated success in increasing the virulence of entomopathogenic fungi. Expression of a scorpion toxin in *M. anisopliae* increased virulence 22-fold towards the tobacco hornworm, *Manduca sexta*, and 9-fold against the mosquito *Aedes aegypti*. Expression of the same toxin in *B. bassiana* was shown to increase its virulence towards a variety of hosts including the pine caterpillar, *Dendrolimus punctatus* [31]. The heterologous expression of toxins, however, has not been without controversy, and the potential for the development of resistance to the toxin remains. We sought, therefore, to develop a different strategy for increasing virulence by using host molecules against the host from which it came [13]. There are several important features of this strategy; first a suitable insect molecule (peptide) must be identified. Numerous insect derived peptides have already been suggested or even employed for insect control [32], [33], [34]. Depending upon the peptide chosen (its distribution and orthology), in principle, various levels of selectivity can be obtained. Although it should be emphasized that such selectivity would need to be verified via experimental analysis, data concerning the spectrum of targets for a number of insect peptides proposed to be used for insect control already exists (see references above). Second, using a host molecule should minimize issues concerning the development of resistance primarily because any mechanism for potentially developing resistance to the host molecule is likely to severely compromise the host. In the case of fire ants, resistance development is even less likely since only queens produce progeny, thus selection occurs within a very small population.

In this report, we improved the virulence of a *B. bassiana* strain to fire ants by expressing a fire ant (neuro-) peptide in the fungal pathogen. Increased virulence in the β-NP expressing fungal strain was noted in both standard and mock mound assays. The increased virulence was specific and no effects were detected against a Lepidopteran hosts (*Galleria mellonella* and *Manduca sexta*), indicating that target-specific virulence can be achieved. This has significant potential for fungal strain improvement and regulatory agencies approval for insect control applications.

Unexpectedly, we noted an altered behavioral pattern in ants infected with the β-NP expressing strain. Rather than forming organized corpse piles as seen in uninfected and wild-type infected ant assays, the dead appeared to remain dispersed throughout the assay chambers. Removal of dead nestmates is thought to limit the potential spread of pathogens, particularly within a social society, and is a common behavior in many ants species. Our observation pointed to altered behavioral effects resulting from application of the β-NP expressing strain on fire ants. These behavioral effects were further probed using the various fungal strains as well as synthetic peptides. Experiments using synthetic peptides indicated that: (1) worker ants have chemosensory perception mechanisms that are able to discriminate between surface peptides, and (2) the β-NP-NH_2_ peptide, but not the non-amidated form, can act as a semiochemical specifically eliciting enhanced necrophoretic behavior. However, although ants killed by the β-NP expressing fungus were moved slower than WT killed or controls, treatment of dead ants with the synthetic β-NP peptide did not show any significant differences in movement times of the dead ants as compared to the controls, i.e. the biological application displayed a phenotype not observed in the application of the synthetic peptide. There are several possible explanations for these results. First, in the biological application, β-NP would be expressed both within the ant (as the infection proceeds) as well as without. Internal expressed β-NP could then have agonistic interactions with the host's amidated peptide and/or receptor(s) or disrupt other host physiological processes that in turn affect (cadaver) recognition cues. Second, the fungal infection may elicit or suppress microbial pathogen/infection detection mechanisms which would not occur when the synthetic peptide is administered externally. Finally, the slower removal times observed using *B. bassiana* β-NP-expressing killed ants could be due to a combination of fungal factors (i.e. fungal produced enzymes, toxins, volatiles, or other compounds) that act in conjunction with the presence of β-NP to affect cadaver removal.

As a facultative parasite, our results expand the realm of examination between *B. bassiana* and their insect targets, which represents a model system in which molecular and cellular dissection of the host-pathogen interaction is beginning to emerge [35], [36], [37], [38], [39]. Our results also open up a new avenue of research with respect to the role and functions of PBAN/pyrokinin peptides in insects, linking them with a complex social behavior. A number of chemical stimuli have previously been reported to act as signals for mediating dead nestmate recognition and removal, i.e. necrophoretic behavior [23], [40]. In particular, increasing concentrations of decomposition products, especially fatty acids such as myristoleic, palmitoleic, oleic, and linoleic acids, appear to be major stimuli in eliciting necrophoretic behavior. It has also been proposed that chemical stimuli that elicit removal of nestmate corpses are present on both live and dead ants, however, live ants contain additional compounds that mask these signals, which are subsequently lost or dissipated upon death. [23]. To date, there are no reports on a peptide acting as a necrophoretic modulating semiochemical. Intriguingly, the draft genome of *S. invicta* has revealed over 400 potential odorant receptor (OR) loci (of which 297 appear to be intact), one of the largest repertoire of such receptors found in insects thus far [41]. Although most ORs are thought to bind hydrophobic and/or volatile compounds and chemicals, it is interesting to speculate that within the *S. invicta* OR set there may be members that can recognize peptides (and discriminate between C-terminal amidated and non-amidated) peptides. This report links β-NP-NH_2_ and necrophoretic behavior. Topical application of PBAN/pyrokinins are known to induce pheromotropic and myotropic activity in live insects [24], however, the full range of their physiological activities remains obscure. Members of the PBAN/pyrokinin family can apparently act as necrophorectic-eliciting cues on dead insects, expanding the potential physiological and sanitary roles of these peptides.
